# Ocular Symptoms in Different Diseases

**Published:** 1881-12

**Authors:** 


### OCULAR SYMPTOMS IN DIFFERENT DISEASES.

Dr. Gorecki, as quoted in the *Glasgow Med. Journal*, has tabulated his views as follows:

Blepharoptosis, or the falling of the upper eyelid, indicates paralysis, complete or incomplete, of the third pair.

Lagophthalmosis, or inability to close completely the palpebral fissure, is a sign of facial hemiplegia, idiopathic or a symptom of cerebral disease.

Stabismus occurring suddenly, and accompanied by diplopia, is most frequently the result of some cerebral affection.

Xanthelasma, a yellow lamina, sometimes met with in the skin of the eyelids, occurs in certain alterations of the liver.

Subconjunctival ecchymoses are frequent in whooping cough, and may sometimes, at the beginning of the complaint, clear up a difficult diagnosis.

Redness of the conjunctiva, watering of the eye, etc., indicate in a child the outbreak of some eruptive fever, particularly measles. The prognosis is favorable if the tears come when the child cries, but fatal if the secretion of tears is arrested.

Spots on the cornea are often the indication of a strumous constitution.

Dilatation of the pupil, or mydriasis, indicates excessive fatigue, the existence of intestinal worms, meningitis in the second stage, or a true amaurosis. The dilatation is most frequently connected with atrophy of the optic nerve. It is seen also during an attack of epilepsy, on coming out of chloroform, after Belladonna poisoning, etc.

Unequal dilatation of the two pupils point to the onset of general progressive paralysis.

Contraction of the pupils is one of the early symptoms of tabes dorsalis. It is met with also at the beginning of meningitis, in Opium poisoning and in Chloral poisoning.

Deformation of the pupil, particularly after the injection of Atropine, indicates an old iritis, in nine cases out of ten, of syphilitic origin, if not depending on some disease of the neighboring parts.

Cataract in subjects under, say forty or fifty, is frequently of diabetic origin, and constitutes soft cataract.

Finally, the opthalmoscope enables us to recognize the retinitis associated with Bright's disease. Retinal hemorrhages, œdema of the retina, and embolism of its central artery, are sometimes met with in organic diseases of the heart. Optic neuritis and perineuritis and atrophy of the disk are symptoms of syphilis, or of tumors in the neighborhood of the cerebellum or the corpora quadrigemina.

